# Structure based design of effective HtpG-derived vaccine antigens against *M. tuberculosis*

**DOI:** 10.3389/fmolb.2022.964645

**Published:** 2022-08-11

**Authors:** Alessia Ruggiero, Han-Gyu Choi, Giovanni Barra, Flavia Squeglia, Young Woo Back, Hwa-Jung Kim, Rita Berisio

**Affiliations:** ^1^ Institute of Biostructures and Bioimaging, IBB, CNR, Napoli, Italy; ^2^ Department of Microbiology and Medical Science, College of Medicine, Chungnam National University, Daejeon, South Korea

**Keywords:** protein structure, vaccine, infectious disease, folding, antigen, chaperone, tuberculosis

## Abstract

Vaccine development against Tuberculosis is a strong need, given the low efficacy of the sole vaccine hitherto used, the Bacillus Calmette–Guérin (BCG) vaccine. The chaperone-like protein HtpG_Mtb_ of *M. tuberculosis* is a large dimeric and multi-domain protein with promising antigenic properties. We here used biophysical and biochemical studies to improve our understanding of the structural basis of HtpG_Mtb_ functional role and immunogenicity, a precious information to engineer improved antigens. We showed that HtpG_Mtb_ is a dimeric nucleotide-binding protein and identified the dimerisation interface on the C-terminal domain of the protein. We also showed that the most immunoreactive regions of the molecule are located on the C-terminal and middle domains of the protein, whereas no role is played by the catalytic N-terminal domain in the elicitation of the immune response. Based on these observations, we experimentally validated our predictions in mice, using a plethora of immunological assays. As an outcome, we designed vaccine antigens with enhanced biophysical properties and ease of production, albeit conserved or enhanced antigenic properties. Our results prove the efficacy of structural vaccinology approaches in improving our understanding of the structural basis of immunogenicity, a precious information to engineer more stable, homogeneous, efficiently produced, and effective vaccine antigens.

## Introduction

Tuberculosis (TB), caused by *Mycobacterium tuberculosis* (Mtb), is still one of the most life threatening infectious diseases (Global tuberculosis report, 2020). The last WHO global TB report (2021) estimates 1.5 million people deaths in 2020, compared to 1.4 million on 2019. This is in full contrast with the target decline of annual TB incidence committed by the WHO End TB strategy (Jeremiah et al., 2022). In most healthy people, the immune system can destroy the bacteria, whereas in some cases, TB infection begins without symptoms before becoming active (latent TB) and can persist for weeks, months or years (Salgame et al., 2015). Up to 10% of people with latent TB develop the disease, through a complex mechanism, which is still not fully understood (Pai et al., 2016). A group of proteins has been identified and characterised, denoted as Resuscitation Promoting Factors (Rpfs), that play a role in Mtb resuscitation through the hydrolysis of its peptidoglycan (Maione et al., 2015; Nikitushkin et al., 2015; Ruggiero et al., 2017, 2016, 2009).

The Stop TB Strategy of WHO aims at controlling TB and, beyond, at eliminating the disease as a global health problem by 2050. However, TB control strategies, albeit ineffective, are a highly cost-effective. Also, delay to diagnosis and inadequate treatment of TB and the development of drug resistance contribute to the severity and mortality of the disease (Bhargava and Bhargava, 2020). Without effective TB vaccines, we are unable to suppress the global TB emergency. Indeed, the only commercially available vaccine against Mtb, the BCG (*M. bovis* Bacillus Calmette Guerin) is not effective in population immunisation (0–80% efficacy in adults and approximately 50% efficacy in children) (Fine, 1989; Sterne et al., 1998). Despite inducing a strong response, this vaccine has proven insufficient to control global TB epidemics, most likely because BCG does not consistently protect against pulmonary TB in adults. A promising strategy in TB vaccine development is to identify vaccine antigens able to boost or improve BCG vaccines (Gupta et al., 2018).

Genomics analysis of mycobacterial strains has revealed that the chromosomal region of difference (RD1) is deleted in all BCG strains and in the naturally-occurring less virulent *M. microti*, but present in all virulent strain of *M. bovis*, all clinical isolates of Mtb, and in the virulent laboratory strain H37Rv (Mahairas et al., 1996; Brodin et al., 2002). RD1 encodes key virulence factors from type VII secretion system ESX-1 (Abdallah et al., 2007), such as the early-secreted antigenic target-6 kDa (ESAT6) and the culture filtrate protein 10 kDa (CFP10). The deletion of RD1 locus has been shown to induce the attenuation of the parental *M. bovis* strain (Lewis et al., 2003; Aagaard et al., 2011; Samten et al., 2011). Importantly, the cytosolic escape of Mtb triggered by ESAT6 results in antigen processing through both class I and class II MHC pathways and the induction of CD8^+^ and CD4^+^ T-cell responses. This explains why BCG, which lacks ESX-1, remains restricted to the phagosome and induces a weaker CD8^+^ T-cell response (Copin et al., 2014; Simeone et al., 2016). A subunit vaccine candidate, consisting of Mtb antigen fusions of Ag85A and ESAT6-CFP-10 (GamTBvac) is currently in phase III clinical evaluation (Vasina et al., 2019; Tkachuk et al., 2020).

We have previously demonstrated that a homolog of the eukaryotic molecular chaperone Hsp90, which we denoted as HtpG_Mtb_, is able to effectively induce dendritic cell (DC) activation (Choi et al., 2017). Also, HtpG_Mtb_ is more effective as antigen in boosting BCG immunization when fused to ESAT6 (Choi et al., 2017). All these data suggested that the DC-activating HtpG_Mtb_ potentiates the protective immunity of T cell antigens (Back et al., 2020).

HtpG_Mtb_ belongs the highly conserved Hsp90 family of protein chaperones. Its homolog from *E. coli* (46% sequence identity) has been shown to play a role in protein folding and stabilisation (Genest et al., 2019). Interestingly, HtpG_Mtb_ is fully conserved among Mtb strains (sequence identities higher than 99%) and highly conserved in pathogenic mycobacteria, like *M. leprae* (Table S1), but it is not encoded in avirulent species like *M. smegmatis*. Consistently, transposon mutagenesis has recently shown that, although the loss of neither HtpG_Mtb_ nor ClpB (another Mtb chaperone) is not lethal to Mtb, the depletion of both chaperones impairs Mtb recovery after exposure to host-like stress (Harnagel et al., 2021).

Structurally, HtpG_Mtb_ adopts a highly stable dimeric structure, a property that is crucial for its functional role as a molecular chaperone (Moreira et al., 2020). Indeed, it is formed by three domains that govern large conformational variations from an open nucleotide-free to a compact ATP-bound state through a clamping mechanism. This mechanism allows HtpG_Mtb_ to bind client proteins to facilitate their folding and then release them once folded, through ATP binding (Meyer et al., 2003; Ali et al., 2006; Shiau et al., 2006; Mader et al., 2020). Structural features of HtpG_Mtb_ also showed that the dimeric state of HtpG_Mtb_ favours ESAT6 dimerisation and hampers ESAT6 cytotoxicity (Moreira et al., 2020).

Using a structural vaccinology approach, we here adopted several biophysics and bioinformatic tools to predict immunogenic epitopes and dissect the contribution of individual domains to folding and antigenicity of HtpG_Mtb_. Upon identification of molecular determinants of antigenicity, we identified subunit vaccine antigens with most optimal properties and experimentally assessed their immunoreactivity using an array of immune assays.

## Methods

### Computational analyses

Homology modelling of HtpG_Mtb_ in its free state was performed as previously reported (Moreira et al., 2020). Namely, consensus-based sequence alignment using the HHpred tool identified the structure of *E. coli* HtpG in the apo state (46% sequence identity on 619 residues, PDB code 2ioq). Using this alignment, the structure was built using the program MODELLER (Webb and Sali, 2017). The structure of HtpG_Mtb_ was also modelled using Artificial Intelligence, and the software Alphafold (Jumper et al., 2021). We generated a three-dimensional model using the Colab server (https://colab.research.google.com/github/sokrypton/ColabFold/blob/main/AlphaFold2.ipynb), which predicts protein structures starting from their sequences using a slightly simplified version of AlphaFold v2.0 that does not consider existing structural templates (Mirdita et al., 2022).

The model of free HtpG_Mtb_ was used for structure-based domain definition, in order to design smaller and improved antigens. To this aim, we also used T cell epitope prediction using by predicting the affinity of epitopes to the MHCII complex, using the software NetMHCIIpan-4.1, which allows predictions of binding to all human MHC class II isotypes (Reynisson et al., 2020). Indeed, the human MHC locus (in humans called HLA for human leukocyte antigens) is extremely polymorphic and encodes thousands of different HLA class II molecules, including HLA-DR, HLA-DP and HLA-DQ molecules. The method is based on artificial neural networks and has been trained on more than 50,000 quantitative peptide-binding measurements covering HLA-DR, HLA-DP, HLA-DQ as well as two murine molecules.

The AlphaFold2.0 model of HtpG_Mtb_ was also used for structure-based B cell epitope prediction, using the software ElliPro (Ponomarenko et al., 2008) and Discotope (Kringelum et al., 2012). Allergenicity was computed using AllergenFP v.1.0 and AllerTOP v2.0 (Dimitrov et al., 2014a; Dimitrov et al., 2014b, p. 0) servers, which categorise amino acids using five E-descriptors of amino acid hydrophobicity, helix-forming propensity, relative abundance of amino acids, and β-strand forming propensity. Proteins are classified by k-nearest neighbour algorithm (kNN, *k* = 1) based on training set known allergens and non-allergens. Toxicity was computed with the ToxinPred protein scanning tool; this method is based on machine learning and quantitative matrix through the detection of residues found in toxins (Gupta et al., 2013).

### Recombinant production of HtpG_Mtb_, isolated domains, and mutated forms HtpG_Mtb__C^F635E^ and HtpG_Mtb__C^triple^

Recombinant production of HtpG_Mtb_ (Rv2299c gene name from Mtb H37Rv) was carried out according to a previously reported procedure (Choi et al., 2017; Moreira et al., 2020). The N-, M-, C- single domains, MC- double domain of HtpG_Mtb_, and the C- mutants were designed based on the homology modelling and the Alphafold2.0 model (Moreira et al., 2020). To this aim, the oligonucleotide primers, Supplementary Table S2, were used to amplify the nucleotide sequence corresponding to these domains by PCR. The PCR product was then cloned into the expression vector pETM-13 (EMBL) or in pET22-b (+). The single point mutant F635E (HtpG_Mtb__C^F635E^) was generated by site-directed mutagenesis of wild-type plasmid using the QuikChange II XL kit (Agilent). The triple mutant carrying mutations F635A, L638A and L639A, HtpG_Mtb__C^triple^, was obtained by using two consecutive PCR cycles (Supplementary Table S2). DNA sequencing confirmed the mutations. To produce a recombinant ESAT6-fused constructs, the PCR products of HtpG_Mtb_, HtpG_Mtb__MC or HtpG_Mtb__C were inserted into the previously produced ESAT-6-containing pET22-b (+) vector. The resulting positive plasmid was used to transform *E. coli* BL21 (DE3) strain. The protein expression was carried out using the transformed cells grown overnight at 37 °C in LB containing 50 μg/ml kanamycin and then inducing them overnight with 0.5 mM IPTG at 18 °C. Cell pellets we resuspended in 30 ml binding buffer (50 mM Tris-HCl pH 8.0, 300 mM NaCl, 5% (v/v) glycerol, 2 mM DTT), added with a protease-inhibitor cocktail (Roche Diagnostic, Italy) and sonicated for 10’. The lysate was cleared by centrifugation at 35,000 × g, and the supernatant was loaded on Ni^2+^-NTA resin (Qiagen) equilibrated with binding buffer. Three washing steps with 10 volumes of binding buffer were performed with increasing concentrations of imidazole. For each purification, the protein was eluted with 150–300 mM of imidazole. A final step of purification was achieved by using size exclusion chromatography on Superdex 75 16/60 (GE Healthcare) (50 mM Tris-HCl pH 8.0, 150 mM NaCl, 2% (v/v) glycerol, 2 mM DTT). In all cases, the proteins eluted in a single peak and were homogeneous as judged by SDS–PAGE analysis. HtpG_Mtb__C^F635E^ and HtpG_Mtb__C^triple^ were produced with the same approach.

### Circular dichroism

All CD spectra were recorded with a Jasco J-810 spectropolarimeter equipped with a Peltier temperature control system (Model PTC-423-S). Molar ellipticity per mean residue [θ] in deg cm^2^·dmol^−1^, was calculated from the equation: [θ] = [θ]_obs_·mrw·(10 *l C*)^−1^, where [θ]_obs_ is the ellipticity measured in degrees, mrw is the mean residue molecular mass (e.g., 111.6 Da for HtpG_Mtb__C), *C* is the protein concentration in mg·ml^−1^ and *l* is the optical path length of the cell in cm. Far-UV measurements (195–260 nm) were carried out at 20°C using a 0.1 cm optical path length cell and a protein concentration of 0.15-0.2 mg ml^−1^. The protein samples were prepared in 20 mM sodium phosphate buffer (pH 7.4). Thermal denaturation studies were performed by recording the CD signal at 222 nm from 20 to 100 C.

### Static light scattering experiments

Purified proteins were analysed by size-exclusion chromatography connected to a triple-angle light scattering detector equipped with a QELS module (quasi-elastic light scattering) for molar mass evaluation. Briefly, 1 mg of protein sample was loaded on a S75 10/300 (GE Healthcare) column, equilibrated in 50 mM Tris-HCl, 150 mM NaCl, 5% (v/v) glycerol, 2 mM DTT (pH 8.0). A constant flow rate (0.5 ml min^−1^) was applied. Elution profiles were detected using a Shodex interferometric refractometer and a MiniDAWN™ Treos light scattering system. Data were analysed by using Astra 5.3.4.14 software (Wyatt Technology, Toulouse, France).

### Isothermal titration calorimetry

The nucleotide binding properties of HtpG_Mtb_ were evaluated by isothermal titration calorimetry (ITC) using a MicroCal iITC200 calorimeter (GE Helthcare, Milan). Prior to binding studies, HtpG_Mtb_ was further purified using ionic exchange chromatography to remove nucleic acid traces and then dialysed in a Hepes-NaCl buffer (pH 7.4) containing 5 mM of MgCl_2_. To evaluate the binding affinity for ADP and AMP-PNP, ITC experiments were performed by adding consecutive injections of 2.0 μl aliquots (at 150 s intervals) of 3 mM nucleotide solutions (1–30 protein:ligand ratio) to the calorimeter cell (0.28 ml). A constant stirring speed of 1,000 rpm was kept during the experiment. In all experiments, protein and ligand solutions were prepared in the same buffer, to minimize the contribution of dilution heat to the measured heat change. Data were analysed using a ‘one set of sites’ binding model. To estimate heats of dilution, separate experiments were carried out by injecting equivalent concentrations of the two nucleotides into the buffer solution. At each injection, the measured heats were subtracted to the binding experiment. Data were analysed using the provided software (Velazquez-Campoy and Freire, 2006).

### Bacterial strains and preparation of *Mycobacterium* spp.

Mtb H37Rv (ATCC 27294) was purchased from American Type Culture Collection (ATCC, Manassas, VA). Mtb was grown in Middlebrook’s 7H9 medium (Difco, Detroit, MI) supplemented with 10% of Middlebrook’s OADC (oleic acid, albumin, dextrose, and catalase) enrichment medium (BBL, Sparks, MD) until late log phase, and frozen at a concentration of 2 × 10^8^ colony forming units (CFU)/ml.

### Animals

Specific pathogen-free female C57BL/6 (H-2K^b^ and I-A^b^) mice and OT-II T-cell receptor (TCR) transgenic mice (C57BL/6 background) were purchased from the Jackson Laboratory (Bar Harbor, ME, United States). The animals were maintained under barrier conditions in a biohazard animal room at the Medical Research Center of Chungnam National University, Daejeon, Korea and were fed a sterile commercial mouse diet and were provided water *ad libitum*. All of animal experiments complied with the ethical and experimental regulations for animal care of Chungnam National University (CNU-00284).

### Immunisation in mice and ELISA analysis for immunoglobulin (Ig) G, IgG1, IgG2b

C57BL/6 mice (*n* = 3 per group) were immunised subcutaneously with BCG or 10 μg HtpG_Mtb_-ESAT6/DDA-MPL. Mice were sacrificed to analyse the level of immunoglobulin at 8 weeks after the protein immunization. Sera were collected from the immunised animals to monitor the antibody response by ELISA. The Nunc ELISA plates were coated with N, M, or C (1 μg/ml). The plates were blocked with TBS containing 3% bovine serum albumin (Sigma-Aldrich, Italy). Sera were added at serial 4-fold dilution (beginning at a 1/10,000 dilution). After washing, HRP-conjugated goat anti-mouse IgG, IgG1 and IgG2b (Thermo Fisher Scientific, Rockford, IL, United States) diluted at 1/2,000 separately in blocking buffer were added. Plates displayed colour by the o-phenylenediamine substrate. Antibody titres were expressed as reciprocal endpoint titres.

### Cell study

Bone marrow-derived DCs and bone marrow-derived macrophages (BMDMs) were generated, cultured and purified as recently described (Choi et al., 2020). DCs (2 × 10^5^ cells/well) were treated with of 10 μg/ml of HtpG_Mtb__N, HtpG_Mtb__M or HtpG_Mtb__C for 24 h. ELISA was used to detecting IL-1β, TNF-α, and IL-12p70 in culture supernatants as described previously (Choi et al., 2017).

For *in vitro* T cell proliferation assay, naïve-T-cells in spleen from OT-II mice were isolated using a MACS column (Miltenyi Biotec, Germany). These T cells were stained with 1 μM CFSE (Invitrogen, San Diego, CA, United States) as previously described (Choi et al., 2017). DCs (2 × 10^5^ cells/well) treated with the OT-II peptides (Peptron, Daejeon, Korea) in the presence of 10 μg/ml of HtpG_Mtb_-E6, HtpG_Mtb__MC-E6, and HtpG_Mtb__C-E6 for 24 h were co-cultured with CFSE-stained CD4^+^ T cells (2 × 10^6^) at DC:T cell ratios of 1:10. After 4 days, each co-cultured T cell batch was stained with PerCP-Cy5.5-conjugated anti-CD4^+^ mAb and analysed by flow cytometry. The supernatants were harvested and assayed for the production of IFN-γ, IL-2, and IL-17 by ELISAs.

### Measurement of intracellular Mtb growth in macrophages

BMDMs (2 × 10^5^ cells/well) were infected in triplicate with Mtb at MOI = 1 for 4 h. Then, the infected BMDMs were treated with amikacin (200 μg/ml) for 2 h, and then washed twice with PBS and this time point was considered day 0. Next, a previously prepared mixture (antigen-activated DCs cocultured with CD4^+^ T cells at a DC:T cell ratio of 1:10 for 3 days) was added to each well, and the plate was incubated for 3 days. DC-activating antigens were LPS (100 ng/ml) Ag85B (10 μg/ml), HtpG (10 μg/ml), HtpG_N (10 μg/ml), HtpG_M (10 μg/ml), HtpG_C (10 μg/ml), HtpG_MC (10 μg/ml), HtpG-E6 (2 μg/ml), HtpG_MC-E6 (2 μg/ml), or HtpG_C-E6 (2 μg/ml). After incubation, the cells were lysed with sterile distilled water. The lysates were serially diluted and plated onto 7H10 agar plates to determine the “input” bacterial numbers. The plates were incubated at 37°C for 3 weeks. At the end of the 3 weeks, plates were taken out and colony forming units (CFUs) were calculated from the number of colonies of Mtb.

### Statistical analysis

All experiments were repeated at least three times with consistent results. Tukey’s multiple comparison test distributions using statistical software (GraphPad Prism Software, version 4.03; GraphPad Software, San Diego, CA) were used to determine statistical differences between samples. The data in the graphs are expressed as the mean ± SEM. Differences with each value of *p < 0.05, **p < 0.01, ***p < 0.001, or ****p < 0.0001 were considered statistically significant.

## Results

**FIGURE 1:**
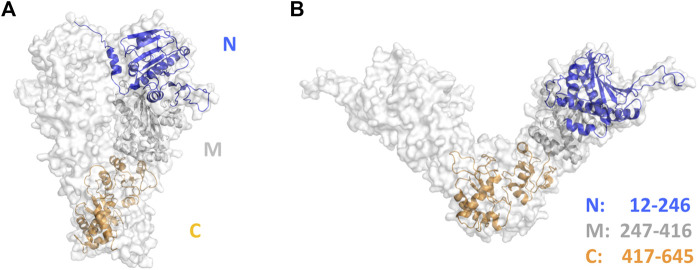
Cartoon and surface representations of **(A)** the AlphaFold2.0 structure of HtpG_Mtb_ and **(B)** its homology model in the ligand-free state. Structure-based domain boundaries of the catalytic N-terminal (N, blue), middle (M, grey) and C-terminal (C, orange) domains are reported with the same colour codes.

In previous work, we have demonstrated that HtpG_Mtb_ is a promising vaccine against tuberculosis (Choi et al., 2017) and adopts a dimeric and highly thermostable ATP-dependent molecular chaperone structure (Moreira et al., 2020). Each chain is composed by three distinct domains, a catalytic, a middle and a C-terminal domain. Here, we provide further biophysical characterisation of HtpG_Mtb_, and exploit structural features of HtpG_Mtb_ (Figure 1) to improve its molecular properties as a vaccine antigen. We employ several bioinformatic tools to predict immunogenic epitopes and to dissect the contribution of individual domains to folding and antigenicity. The experimental assessment of our predictions allows us to identify the most immunoreactive regions of the molecule for vaccine development and to engineer an improved antigen.

### HtpG_Mtb_ is a stable dimer that binds both ADP and AMP-PNP

We have recently homology modelled the structure of HtpG_Mtb_ in all functional states, including a substrate-free, an ADP- and an ATP-bound state (Moreira et al., 2020). We here confirm the chaperone-like structure of HtpG_Mtb_ using the most recent artificial intelligence strategy of structure prediction, Alphafold2.0, which has been shown to be capable of predicting protein structures to near experimental accuracy (Jumper et al., 2021). The structural description of HtpG_Mtb_ according to Alphafold2.0 has a close resemblance with the model we previously described as ATP-bound (Figure 1) (Moreira et al., 2020). Different from the ligand free form, a highly compact structure characterises the ATP-bound and the AlphaFold2.0 model structure, which also presents the swapping of the N-terminal strands (Figure 1A).

**FIGURE 2:**
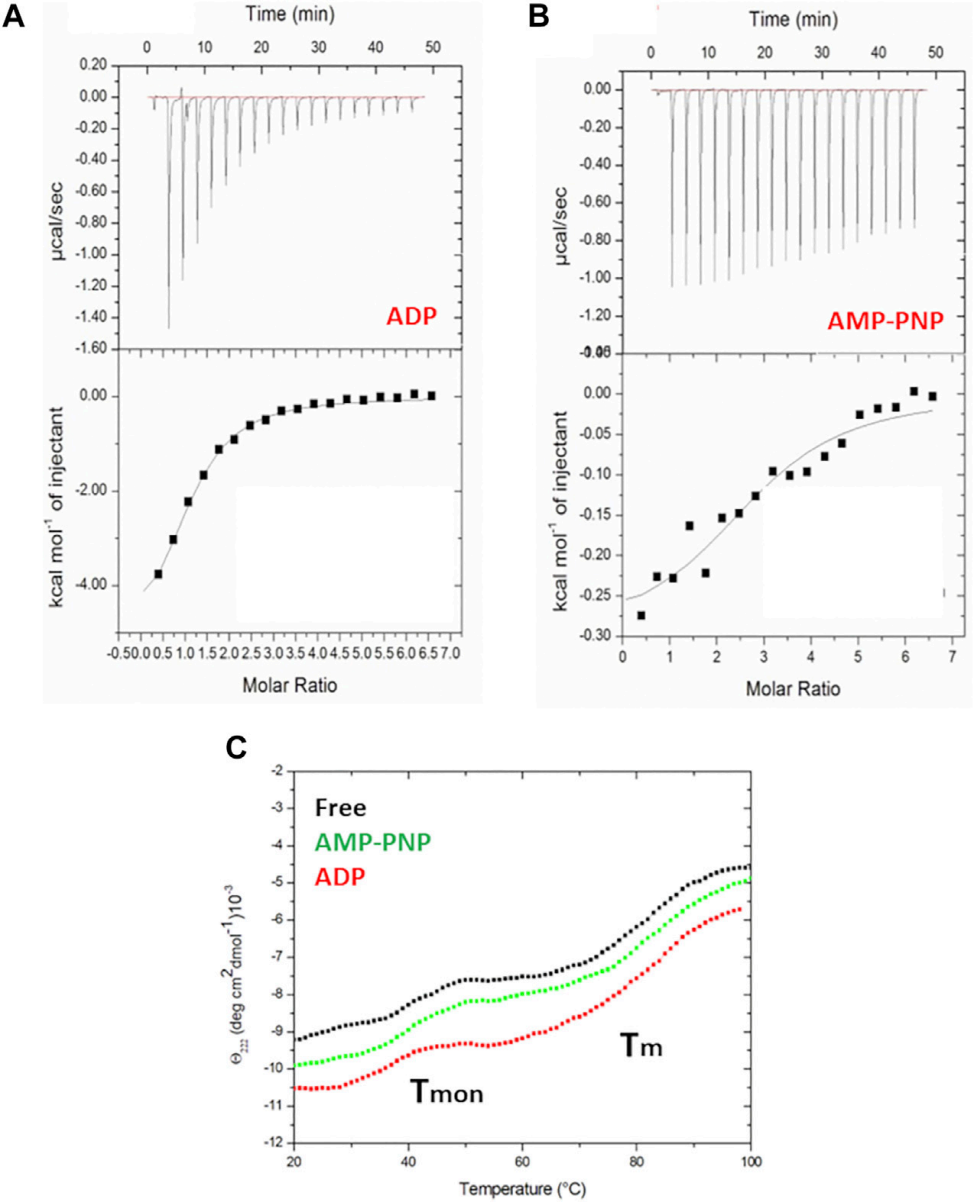
Isothermal titration calorimetry of HtpG_Mtb_ wild type with ADP **(A)** and AMP-PNP **(B)**. Top panels report raw data for the titrations at 22°C, whereas bottom panels report integrated heats of binding obtained from the raw data after subtracting the heats of dilution. The solid lines in bottom panels represent the best curve fits to the experimental data using the ‘one set of sites’ model from MicroCal Origin. **(C)** Thermal denaturation curves of HtpG_Mtb_ with and without nucleotides (AMP and AMP-PNP), monitored at 222 nm in 20 mM sodium phosphate (pH 7.4).

Consistent with the role and the structure of HtpG_Mtb_ as a chaperone molecule, we observed, using Isothermal Titration Calorimetry (ITC) that HtpG_Mtb_ is able bind both ADP and AMP-PNP, a non-hydrolysable analogue of ATP (Figure 2). For ADP, the measured experimental values, averaged over two independent experiments, show a single site of binding (Table 1) and a dissociation constant K_d_ of 34.0 (±0.1) μM. In the case of AMP-PNP we measured a comparable K_d_, of 45.4 (±0.2) μM, but associated to two binding sites (Table 1). Two AMP-PNP sites were also found for Hsp90 and the Hsp110 chaperone family, although the location of the second AMP-PNP remains undetermined (Wawrzynow et al., 1996; Garnier et al., 2002). In addition, thermodynamic parameters determined by ITC for HtpG_Mtb_ are in accordance with those observed for the homologous Hsp90 (Garnier et al., 2002; Zhang et al., 2015). Indeed, as observed for Hsp90, the unfavourable entropy value in the AMP-PNP binding experiment (Table 1) is consistent with the large conformational changes of HtpG_Mtb_ associated with the binding event (Figure 1) (Zhang et al., 2015).

**TABLE 1:** Thermodynamic binding parameters of HtpG_Mtb_ obtained with ADP and AMP-PNP titration by ITC at 22 °C and pH 7.4.

**Parameters**	**ADP**	**AMP-PNP**
**K**_**a**_ **x 10** ^**4**^ **(M** ^**−1**^ **)** ^a^	2.94 ± 0.32	2.16 ± 0.93
**ΔH (kcal mol** ^**−1**^ **)**	−5.66 ± 0.32	–0.30 ± 0.04
**ΔS (cal mol** ^**−1**^ **deg** ^**−1**^ **)**	1.28	18.80
**N**	1	2

a K_a_ is the stoichiometric equilibrium constant.

The different structural features of HtpG_Mtb_ in its different functional states prompted us to investigate its unfolding properties in the presence of ADP and AMP-PNP. As shown in Figure 2C, the presence of either ADP or AMP-PNP does not affect the thermal unfolding curves of HtpG_Mtb_, recorded by following the CD signal at 222 nm as a function of temperature. As observed for the free enzyme, thermal unfolding curves at pH 7.4 reveal that two transitions exist, the first (T_mon_) close to 40°C corresponding to the monomerisation of the enzyme, and the second due to protein unfolding, T_m_.

### HtpG_Mtb_ dimerises through a four-helix bundle in the C terminal domain

**FIGURE 3:**
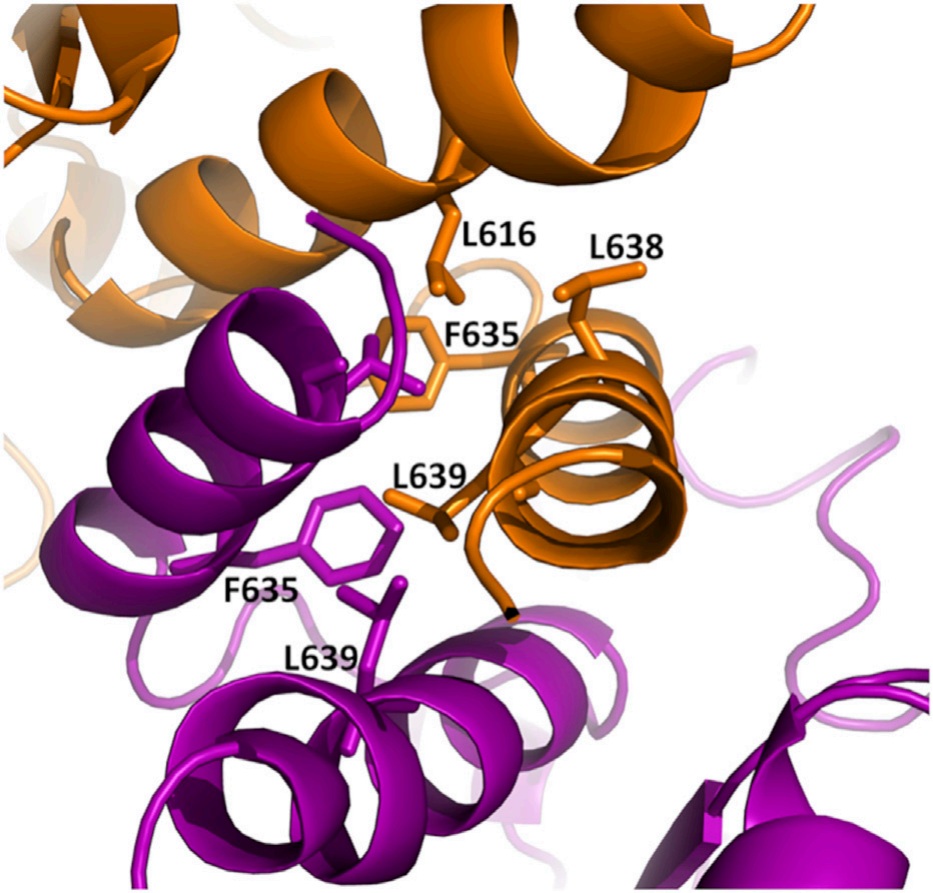
Cartoon representation of the dimerization interface of HtpG_Mtb_. The two chains are represented in orange and prune. Key hydrophobic residues of the four-helix-bundle hydrophobic core are drawn in stick representation.

**FIGURE 4:**
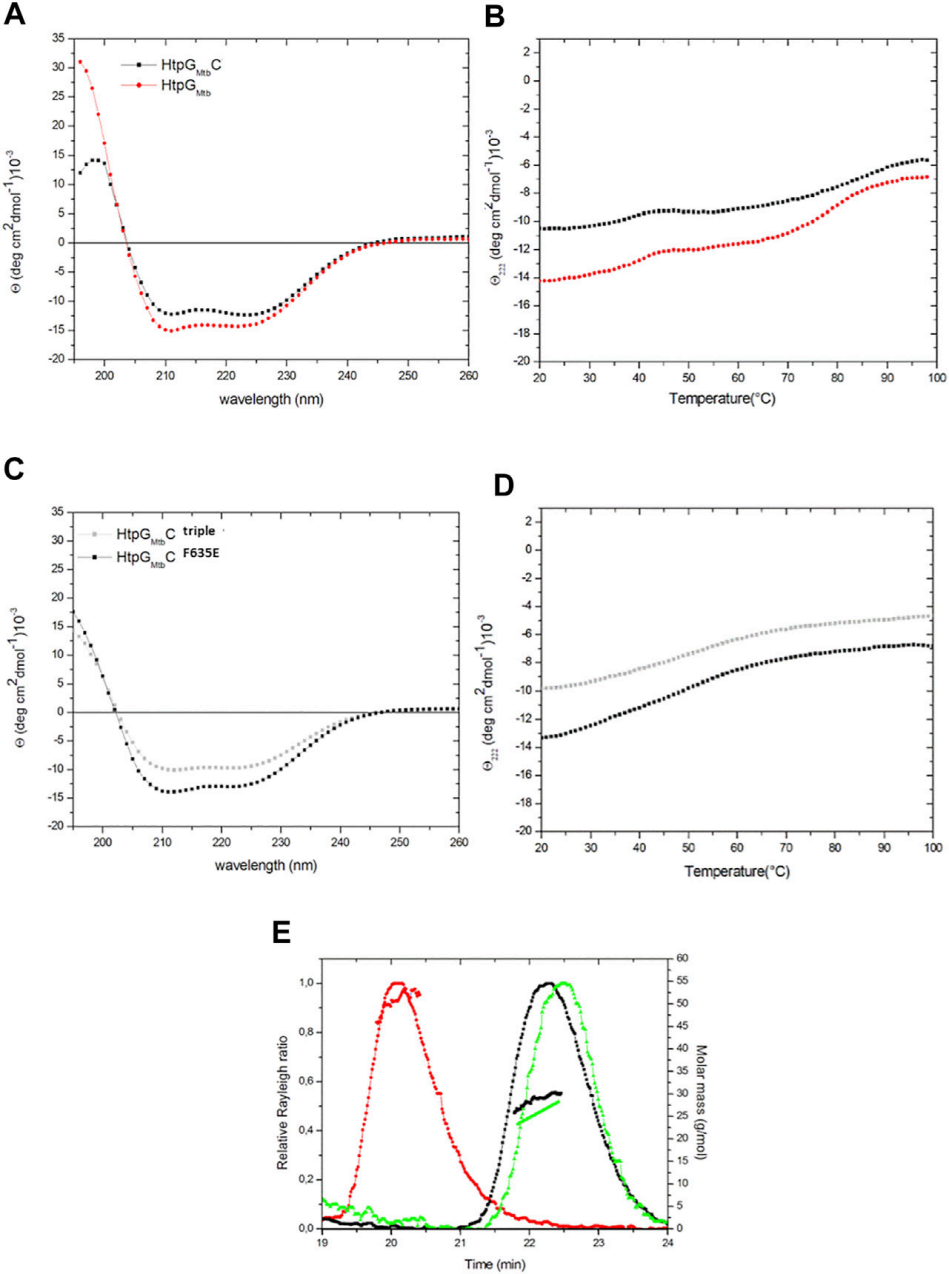
Superposition of CD spectra **(A)** and thermal unfolding at 222 nm **(B)** of HtpG_Mtb_ and HtpG_Mtb__C, measured in 20 mM sodium phosphate (pH 7.4). **(C)** Superposition of CD spectra and **(D)** thermal unfolding at 222 nm of HtpG_Mtb__C mutants in 20 mM sodium phosphate (pH 7.4). **(E)** Analytical SEC-LS of HtpG_Mtb__C wild-type (red) and its variants HtpG_Mtb__C^F635E^ (black) and HtpG_Mtb__C^triple^ (green). The curves represent the Rayleigh ratio (left scale) against the retention time. Molar mass values are reported in the right scale.

As suggested by its structure, the dimerisation of HtpG_Mtb_ is mostly governed by its C-terminal domain, embedding residues 417–645 (Figure 1). Indeed, C-terminal domains of each chain form a highly stable structure, with a gain in free energy of solvation, Δ^i^G = −22.2 kcal/mol, as computed by PISA (Krissinel and Henrick, 2005). Analysis of the dimer interface shows that key to dimerisation are the two C-terminal α helices, embedding residues 609–624 (SLAETAELLYGTALLA) and 632–642 (PARFAELLAER) (Figure 3). These helices form a compact 4-helix bundle, with some hydrophobic residues, which are crucial for dimerisation. Among those, F635 forms hydrophobic interactions with L639 of the adjacent chain. Also, L638 forms intra-chain hydrophobic interactions with L616 of helix 609–624 (Figure 3). Therefore, we decided to recombinantly produce the C-terminal domain (HtpG_Mtb__C) and perform 1) a single disruptive mutation of F635 to glutamic acid (HtpG_Mtb__C^F635E^) and 2) a triple mutant with F635, L638 and L639 mutated to alanine (HtpG_Mtb__C^triple^). Structural properties in solution were analysed using far-UV CD spectroscopy and analytical size-exclusion chromatography (SEC) with multi-angle light scattering (MALS). Both the CD spectrum and the thermal unfolding curves of HtpG_Mtb__C are nearly superposable with that of the entire HtpG_Mtb_, with a monomerisation transition (T_mon_) close to 40°C and an unfolding transition at T_m_ = 85°C (Figures 4A,B). Completely different unfolding curves are observed for the mutants HtpG_Mtb__C^F635E^ and HtpG_Mtb__C^triple^, where unfolding profiles present a single cooperative transition (Figures 4C,D). Consistently, the on-line measurement of the intensity of the Rayleigh scattering as a function of the angle produced MW values for the mutants. We have found a monomeric state for both HtpG_Mtb__C^F635E^ (MW 27.5 ± 0.27 kDa) and HtpG_Mtb__C^triple^ MW (29.4 ± 0.15 kDa) (Figure 4E, black and green, respectively), whereas HtpG_Mtb__C appears dimeric, with MW (53.8 ± 0.3 kDa), Figure 4E (red). These data identify the molecular determinants responsible for HtpG_Mtb_ dimeric state, a crucial feature for the functional role of this protein as a chaperone.

### HtpG_Mtb_ as a vaccine antigen: prediction of T cell and B cell epitopes

We have previously demonstrated that HtpG_Mtb_ (Rv2299c) activates dendritic cells (DCs), which can induce T cell differentiation, resulting in inhibition of intracellular Mtb growth in macrophages. Also, fusion of ESAT6 to the C-terminal end of HtpG_Mtb_ results in significant enhancement of vaccine efficacy as a BCG booster (Choi et al., 2017). DC take up either endogenous antigens or pathogens, to generate peptide complexes with the major histocompatibility complex (MHC), to eventually interact with and stimulate T lymphocytes. MHCII antigen presentation typically occurs for exogenous antigens whereas MHCI is generally limited to endogenous antigens (Blum et al., 2013).

Given the large size of HtpG_Mtb_ and its easy proteolytic degradation (data not shown), we aimed to identify immunoreactive regions of the protein, to develop a more stable and effective antigen. Therefore, we performed epitope prediction to identify those epitopes with high affinity of binding to MHCII, as the identification of peptides that bind to the MHCII molecules is of great importance for the design of new vaccines and immunotherapies. We also computed epitope predicted affinity to the MHCII complex, including HLA-DR, HLA-DP and HLA-DQ molecules, using the software NetMHCIIpan-4.1 (Reynisson et al., 2020, p. 1). This analysis identified 47 strong epitopes, having predicted IC50 lower than 50 nM, of which 40 with HLA-DR molecules and 7 with HLA-DP (Table 2, Supplementary Table S3). All epitopes predicted to bind HLA-DQ molecules presented a predicted IC50 higher than 300 nM. Importantly, none of the identified epitopes were predicted to be either allergenic or toxic, using the software AllergenFP v1.0 (Dimitrov et al., 2014b) and ToxinPred (Gupta et al., 2013), respectively.

**TABLE 2:** Predicted number of strong binding MHC II epitopes, derived using NetMHCIIpan4.1 (IC50 cut-off of ≤ 50 nM). Epitope sequences are reported in Supplementary Table S3.

HLA II Alleles	Domain N	Domain M	Domain C	Total
HLA-DRB1_0101	1	2	2	5
HLA-DRB1_0102	1	2	2	5
HLA-DRB1_0301	1	1	0	2
HLA-DRB1_0305	1	0	0	1
HLA-DRB1_0404	1	0	0	1
HLA-DRB1_0701	1	0	0	1
HLA-DRB1_0901	0	0	2	2
HLA-DRB1_1,001	0	2	1	3
HLA-DRB1_1,101	2	1	1	4
HLA-DRB1_1,104	2	1	2	5
HLA-DRB1_1,301	1	1	0	2
HLA-DRB1_1,302	0	0	1	1
HLA-DRB5_0101	0	0	1	1
HLA-DRB3_0202	0	0	1	1
HLA-DRB3_0101	1	0	0	1
HLA-DRB1_1,601	0	0	1	1
HLA-DRB1_1,501	2	0	1	3
HLA-DRB1_1,402	0	0	1	1
HLA-DPA10103-DPB10201	0	1	2	3
HLA-DPA10103-DPB10401	0	1	1	2
HLA-DPA10103-DPB12301	0	1	1	2
	**14**	**13**	**20**	**47**

In addition to these analyses, reliable modelling allowed us a precise dissection of domain boundaries. Namely, N-terminal (N), middle (M) and C-terminal (C) domains include residues 12–246, 247–416 and 417–645, respectively, (Figure 1). Using this information, we could assess that most of the predicted epitopes, 20 over 47, belonged to domain C, 14 to domain N, 13 to domain M (Table 2, Supplementary Table S3). Using these data and precise domain boundaries of HtpG_Mtb_ we aimed at designing smaller and more stable vaccine antigens, albeit with similar or enhanced antigenic properties, compared to the entire HtpG_Mtb_. For an experimental assessment of antigenic properties of designed antigens, we recombinantly produced isolated domains, as explained below.

We also performed epitope predictions to identify possible B cell epitopes. Given the well-known correlation between antigenicity, solvent accessibility, and flexibility of antigenic regions in proteins, the knowledge of the three-dimensional structure of an antigen helps in the reliability of antigen predictions. Given a three-dimensional structure, B cell epitopes can be classified as linear, made of single continuous stretch of amino acids or conformational/discontinuous, where residues are distantly separated in the sequence and brought into physical proximity by protein folding. Therefore, we used the AlphaFold2.0 model for structure-based epitope predictions, using the software ElliPro (Ponomarenko et al., 2008) and Discotope (Kringelum et al., 2012), detecting linear and discontinuous epitopes, respectively.

Antigen prediction with ElliPro detected three strong B cell epitope peptides (score > 0.80), two of which are in the C-terminal domain of the enzyme, and one on the connecting loop between the N and M domains (Table 3). None of the identified epitopes were predicted to be either allergenic or toxic (Table 3), using the software AllergenFP v1.0 (Dimitrov et al., 2014b, p. 0) and Toxin-Pred (Gupta et al., 2013), respectively. These data predict that the M and C domains of HtpG_Mtb_ can stimulate a B cell response. An added value of the C domain is its dimeric organisation, which allows for multiple presentation of B cell antigens. Discotope analysis also identified best discontinuous epitopes, mostly located either on the M or the C domain (Supplementary Figure S1).

**TABLE 3:** Structure based predicted B-cell epitopes, according to ElliPro (score threshold 0.80).

Antigenic region	Domain	Residues	ElliPro score	AllergenFP	Toxin-Pred
WPIRMDVERRTPASQEEGGEGGEETVTIETETLNSM	N-M	212–247	0.83	Non-allergen	Non-toxin
ELNPSHPLVTGLRQAHQDRADDAEKSLAETA	C	584–614	0.82	Non-allergen	Non-toxin
AKGEVDLSSEEDTSEAEREERQKEFADLLTWLQETLSDHVKEVRLSTRLTESP	C	499–551	0.81	Non-allergen	Non-toxin

### Immunoreactivity of the recombinant HtpG_Mtb_ domains (N, M, C)

**FIGURE 5:**
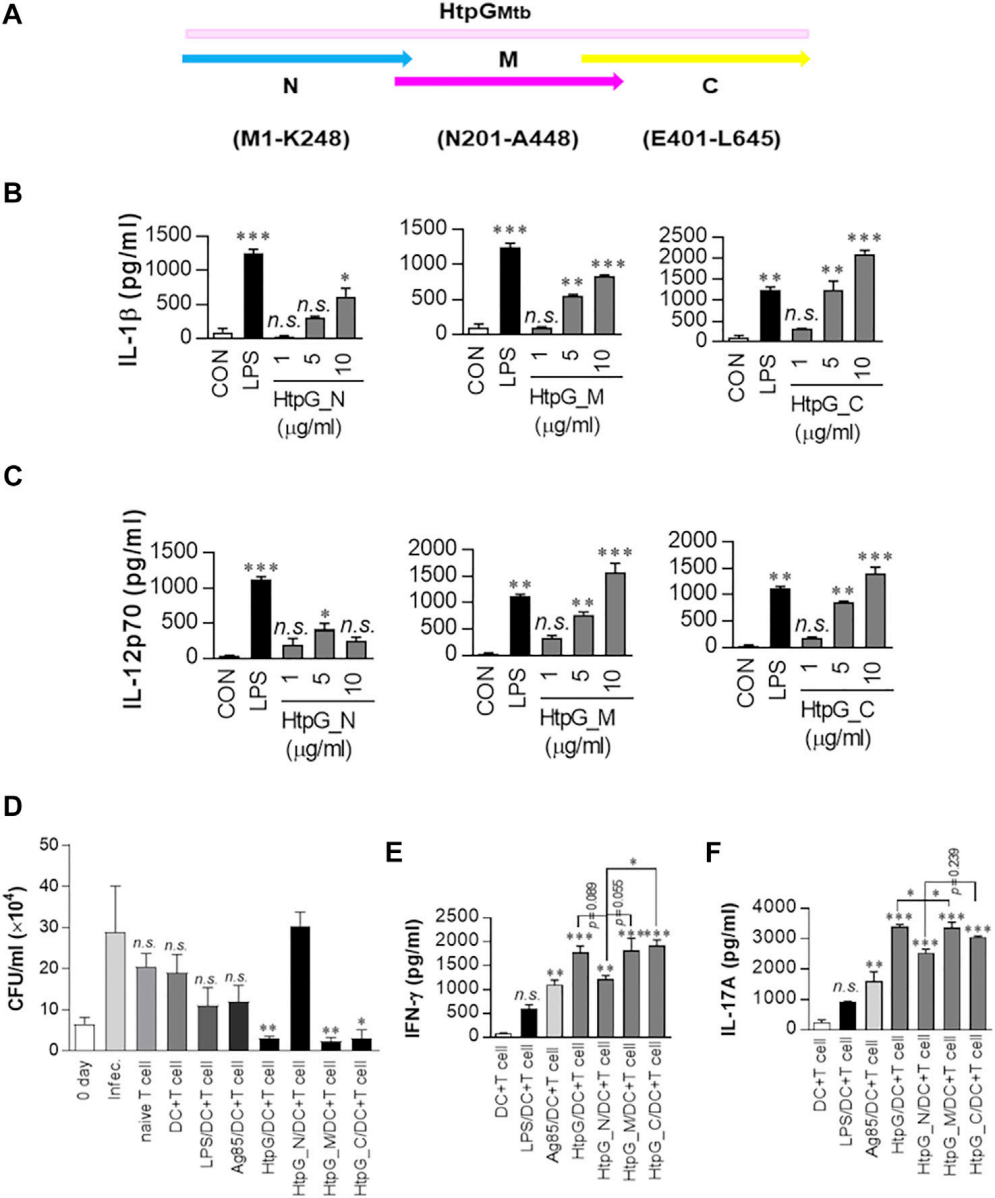
Immunoreactivity of the recombinant HtpG_Mtb_ domains (N, M, **(C)**. **(A)** Sequence borders of each domain of HtpG_Mtb_. **(B)** IL-1β and **(C)** IL-12p70 levels in the culture medium were measured by ELISA. Before the ELISA assay, DCs were activated with the indicated concentration of HtpG_Mtb__N, HtpG_Mtb__M, HtpG_Mtb__C, or LPS (100 ng/ml) for 24 h. Data are presented as mean ± SEM (*n* = 5); *p < 0.05, **p < 0.01, and ***p < 0.001 for treated compared to untreated DCs (CON). *n. s.*: no significant difference. **(D)** Intracellular Mtb growth in BMDMs (reported as colony forming units (CFU)/ml) determined at time point 0 (0 days) and 3 days after coculturing with T cells or without T cells (control). The data shown are the mean values ± SD (*n* = 3); *p < 0.05, or **p < 0.01 for BMDMs cocultured with T cells compared to infection group. *n. s*.: no significant difference. Prior to the assay, naïve T cells or T cells activated by unstimulated DCs, LPS-, Ag85B-, HtpG_Mtb_-, HtpG_Mtb__N-, HtpG_Mtb__M-, or HtpG_Mtb__C-stimulated DCs (DC:T cell ratio of 1:10 for 3 days) were cocultured with BMDMs infected with Mtb. **(E,F)** Cytokine levels in culture supernatants measured by ELISA. The data shown are the mean values ± SD (*n* = 3); *p < 0.05, **p < 0.01, or ***p < 0.001 for antigen-treated DC cocultured with T cells, compared to untreated DC cocultured with T cells. *n. s*.: no significant difference.

For an experimental assessment of the immunoreactivity of the isolated epitope-containing domains, we analysed their effect on bone marrow-derived dendritic cells (BMDCs). Since we detected epitopes also in the connecting loops between domains (Table 3), we produced recombinant domains having a short overlap to preserve those loops (Figure 5A). As a result, we observed that all domains stimulated BMDCs to secret significant IL-1β production compared to unstimulated cells, with a concentration-dependent response (from 1 to 10 μg/ml of isolated domains, Figure 5B). At all tested concentrations, IL-1β production increased going from the N- to the C-domain, with this latter stimulating nearly 4-fold higher IL-1β production compared to the N domain (Figure 5B). Production of IL-12p70 in DCs stimulated cells was also analysed, as it is a critical cytokine for Th1 differentiation. As shown in Figure 5C, when DC are stimulated with either M- or C-domains, we observe a clear dose-dependent IL-12p70 production, whereas scarce production characterises DC stimulated with the M- domain (Figure 5C).

To assess the effective role of DC stimulated by isolated domains to control Mtb, we used naïve T cells from uninfected mice and activated them by coculturing with each antigen matured DCs for 72 h. Following this incubation, we added activated T cells to Mtb-infected bone marrow-derived macrophages (BMDMs). As shown in Figure 5D, T cells activated by full-length HtpG_Mtb_, M-, and C-domain-matured DCs significantly inhibited the Mtb growth, compared to infection control. By contrast, N-domain matured DC cells did not induce T cells with anti-mycobactericidal activity. Importantly, T cells activated by DCs after stimulation with either LPS or the Mtb antigen Ag85B, used as controls, induced much lower and barely significant Mtb growth inhibition, compared to that observed upon stimulation by either HtpG_Mtb_, M or C domains. We also measured IFN-γ and IL-17 levels in the supernatants of the co-cultured cells (Figures 5E,F). Also in this case, the production of these cytokines was significantly higher when Mtb-infected macrophages were treated with T cells activated by either HtpG_Mtb_, M- or C-maturated DCs (Figure 5E).

**FIGURE 6:**
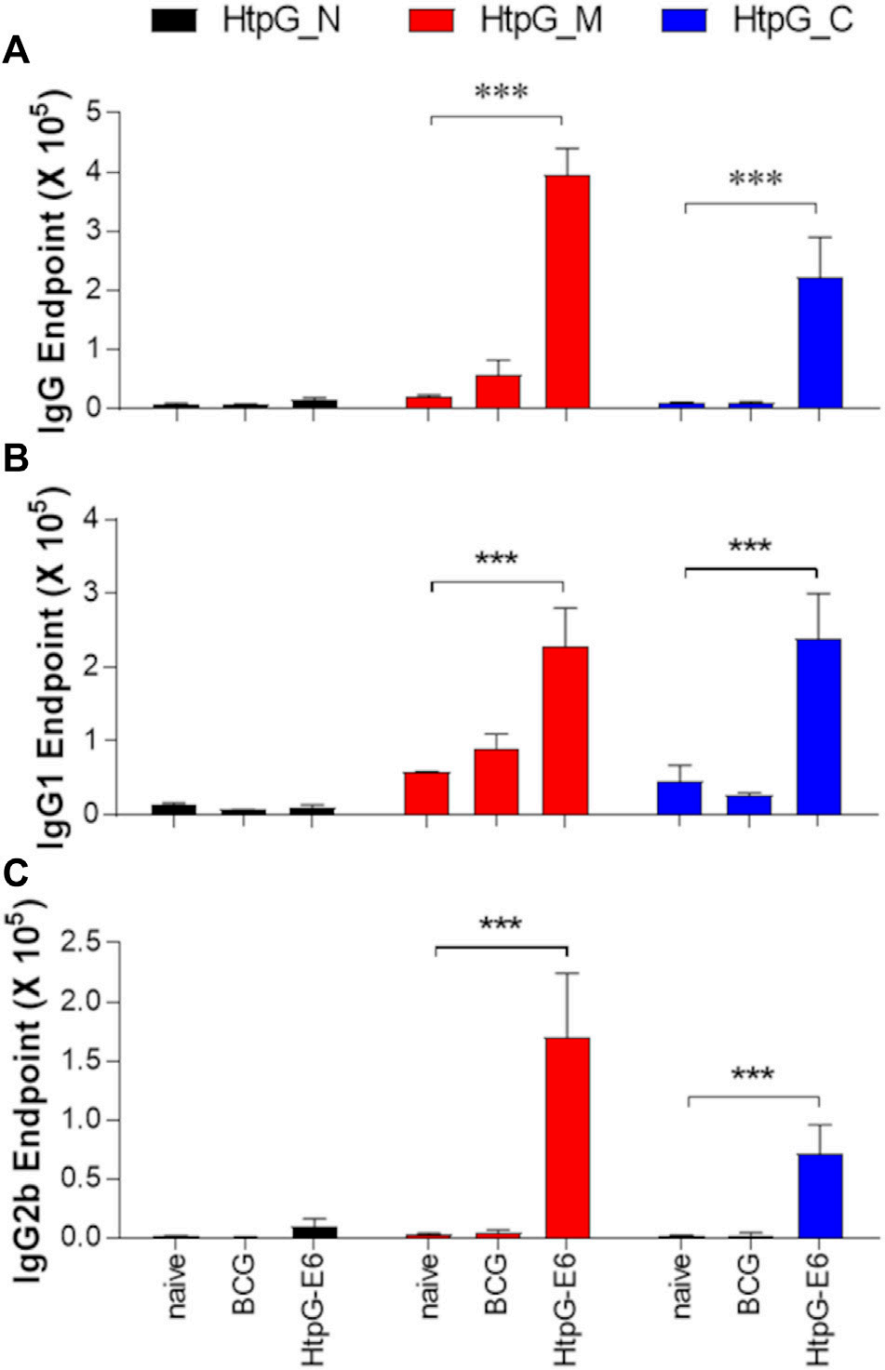
Antibody responses against HtpG_Mtb_ N, M, and C domains, measured in the mice serum 6 weeks after the last immunisation or 10 weeks after BCG injection. Data are presented as mean ± SEM (n = 3); ***p < 0.001.

To experimentally assess the ability of HtpG_Mtb_ to induce a B cell response, we measured the antibody titres against N, M and C domains in mice, after immunisation with either HtpG_Mtb_-ESAT6 fusion protein or BCG (Figure 6). In the case of BCG-injected mice, we observed no specific antibody responses to either N, M or C domains. By contrast, in HtpG_Mtb_-ESAT6 immunised mice, there is a strong production of total IgG-, IgG1-, and IgG2b against M and C domains whereas the N domain induced no antibody response (Figure 6). Taken together, these data suggest that the antigenic activity of HtpG_Mtb_, both as T cell and B cell stimulator, is mainly located in HtpG_M- and HtpG_C-domain regions.

### Immunogenicity of the domains of HtpG_Mtb_ fused to ESAT6

**FIGURE 7:**
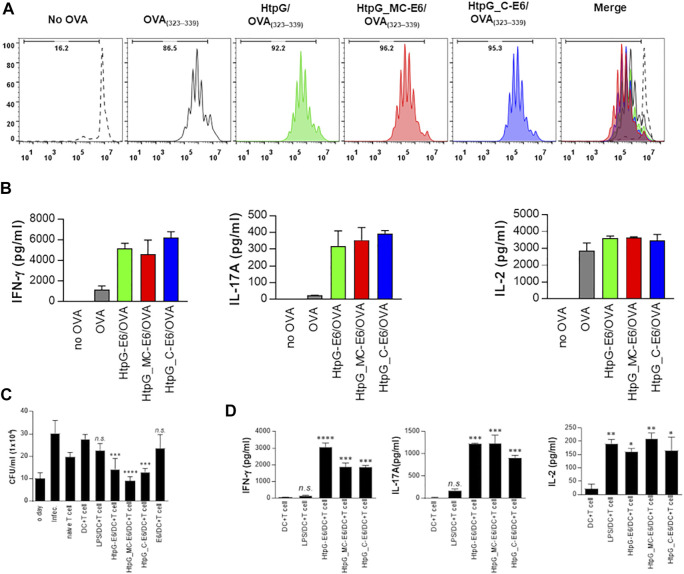
Immunogenicity of the domains of HtpG_Mtb_ fused to ESAT6 (E6). **(A)** Proliferation of OT-II^+^ T cells assessed by flow cytometry: Transgenic OVA-specific CD4^+^ T cells were isolated, stained with CFSE, and cocultured for 96 h with DCs treated with HtpG_Mtb_-E6 (2 μg/ml), HtpG_Mtb__MC-E6 (2 μg/ml), or HtpG_Mtb__C-E6 (2 μg/ml), then pulsed with OVA_323–339_ (1 μg/ml) for OVA-specific CD4^+^ T cells, respectively. T cells only and T cells cocultured with untreated DCs served as controls. **(B)** IFN-γ, IL-2, and IL-17 levels in the culture supernatants harvested after 24 h and assayed by ELISA. **(C,D)** Naïve T cells or T cells activated by unstimulated DCs, LPS-, HtpG_Mtb_-E6-, HtpG_Mtb__MC-E6-, or HtpG_Mtb__C-E6-stimulated DCs (DC:T cell ratio of 1:10 for 3 days) were cocultured with BMDMs infected with Mtb. Intracellular Mtb growth in BMDMs **(C)** was determined at time points 0 (day 0) and 3 days after coculturing with T cells or without T cells (control). The cytokine levels in culture supernatants were measured by ELISA **(D)**. The data shown are the mean values ± SD (*n* = 3); *p < 0.05, **p < 0.01, ***p < 0.001, or ****p < 0.0001 for antigen-treated DC cocultured with T cells, compared to untreated DC cocultured with T cell. *n. s*.: no significant difference.

We have demonstrated the promising vaccine efficacy of the HtpG_Mtb_-ESAT6 fusion protein as a BCG booster (Choi et al., 2017). Based on our results showing that C domains and, to a minor extent, M domains are the immunoreactive regions of the molecule, we prepared fusion proteins by coupling ESAT6 to the C domain (HtpG_Mtb__C-ESAT6) and the MC domain (HtpG_Mtb__MC-ESAT6); the ESAT6-fused entire protein (HtpG_Mtb__ESAT6) was used as a control. Next, to evaluate the activity of the fusion proteins on the interaction between DCs and T cells, we performed a syngeneic *in vitro* T-cell proliferation assay using OT-II TCR transgenic CD4^+^ T cells. DCs pulsed with the ovalbumin 323–339 peptide (OVA_323–339_) were cocultured with transgenic CFSE-labelled OVA-specific CD4^+^ T cells for 72 h. All DCs treated with fusion proteins induced significant T-cell proliferation, compared to untreated DCs (Figure 7A). Furthermore, naïve CD4^+^ T cells primed with DCs treated with each fusion protein produced significantly greater IFN-γ and IL-17 amounts than those with untreated DCs (Figure 7B). However, the amount of IL-2 was similar, as IL-2 was used as a research tool to measure proliferation of T cell populations for *in vitro* assays. Finally, we repeated the same experiment of Figure 5D to determine the antimycobacterial activity of the fusion proteins. As shown in Figure 7C, T cells activated by DCs matured with three fusion proteins significantly inhibited Mtb growth in macrophages when compared to infection control. Interestingly, HtpG_Mtb__MC-ESAT6 showed a higher inhibition effect than the full-length HtpG_Mtb_-ESAT6 or HtpG_Mtb__C-ESAT6. As controls, naïve T cells only or T cells activated by untreated-DCs, LPS-treated DCs, and ESAT6-treated DCs did not show any significant inhibition, Figure 7C. Also, the production of IFN-γ, IL-17, and IL-2 was significantly higher in Mtb-infected macrophages co-cultured with T cells activated by DCs treated with all tested fusion proteins than those of untreated-DCs (Figure 7D). Taken together, our data suggest that either HtpG_Mtb__MC-ESAT6 or HtpG_Mtb__C-ESAT6 present similar or better immunoreactive performances than the full-length HtpG_Mtb_-ESAT6.

## Discussion

There is currently no licensed vaccine for *M. tuberculosis*; however, due to the pathogens’ intrinsic antimicrobial resistance and the mortality rate associated with the disease, there is a pressing need for one. Numerous vaccine candidates are currently in clinical trials, including mycobacterial killed, whole-cell, or extract vaccine candidates (Vaccae, MIP, DAR-901, and RUTI), live-attenuated mycobacterial vaccine candidates (VPM1002, BCG revaccination, and MTBVAC), recombinant live-attenuated or replication-deficient virus-vectored candidates expressing *M. tuberculosis* proteins (TB/FLU-04L, Ad5Ag85A, and ChAdOx1.85A/MVA85A). In addition to these, recombinant fusion proteins are considered as safer and are currently both in phase I (H56) and phase III (GamTBvac) clinical evaluation (Sable et al., 2019). Importantly, there is growing interest in recombinant multivalent vaccines where the fused proteins elicit effective and simultaneous immune responses at different levels (Choi et al., 2017, Choi et al., 2020; Kramarska et al., 2021). In this framework, structural vaccinology is a strong tool to design and develop tailored recombinant antigens. This methodology rationally aims to generate an effective vaccine antigen, combining biochemistry, molecular biology and structural determination methods with computational tools including molecular modelling, epitope prediction, prediction of epitope binding affinity to the Major histocompatibility complex (MHC) (Schijns et al., 2021).

In this work, we based on our previous identification of a vaccine antigen, HtpG_Mtb_, which can stimulate dendritic cells, the most efficient antigen-presenting cells (Choi et al., 2017) (Moreira et al., 2020). We showed that, consistent with other chaperones (Genest et al., 2019), HtpG_Mtb_ is a dimeric nucleotide-binding protein. Using molecular modelling and experimental mutational assessment, we also identified the dimerisation interface, located on the protein C-terminal domain. The gathered structural information was the starting point for computational and experimental studies to dissect molecular determinants of HtpG_Mtb_ antigenicity. Indeed, the elucidation of the protein regions responsible for its immunoreactivity is an important tool for the development of improved antigens (Schijns et al., 2021). Epitope predictions revealed that the most immunoreactive region of the molecule is located on the C-terminal and middle domains. By contrast, the catalytic and nucleotide-binding N-terminal domain plays no role in elicitation of the immune response. Then, we experimentally validated our predictions, using a plethora of immune assays.

As an outcome, we designed vaccine antigens with enhanced biophysical properties albeit conserved or enhanced antigenic properties. Importantly, we produced an antigen upon fusion of middle and C-terminal domains of HtpG_Mtb_ with ESAT6 that possesses a higher antimycobacterial activity than the fusion protein embedding the entire HtpG_Mtb_. Our results prove the efficacy of structural vaccinology approaches in improving our understanding of the structural basis for immunogenicity, a precious information to engineer more stable, homogeneous, efficiently produced vaccine and effective antigens.

## Data Availability

The raw data supporting the conclusions of this article will be made available by the authors, without undue reservation.
