# Supplementary material for: Structure based design of effective HtpG-derived vaccine antigens against M. tuberculosis
Source: Front Mol Biosci. 2022 Aug 11;9:964645. doi: 10.3389/fmolb.2022.964645 (PMC9403545; doi:10.3389/fmolb.2022.964645)
Supplement: Supplementary file 1 [file Table1.docx]

Supplementary Material

# Supplementary Tables

**Table S1.**  **Sequence conservation of HtpG_Mtb_ in *mycobacteria****

| **Accession code** | [**Species**](https://blast.ncbi.nlm.nih.gov/Blast.cgi) | [**Query Cover**](https://blast.ncbi.nlm.nih.gov/Blast.cgi?CMD=Get&ADV_VIEW=yes&ADV_VIEW=on&ALIGNMENTS=100&ALIGNMENT_VIEW=Pairwise&CDD_RID=CDAEDX7U01R&CDD_SEARCH_STATE=0&CONFIG_DESCR=ClustMemNbr,ClustComn,Ds,Sc,Ms,Ts,Cov,Eval,Idnt,AccLen,Acc&DATABASE_SORT=0&DESCRIPTIONS=100&DYNAMIC_FORMAT=on&FIRST_QUERY_NUM=0&FORMAT_NUM_ORG=1&FORMAT_OBJECT=Alignment&FORMAT_PAGE_TARGET=&FORMAT_TYPE=HTML&GET_SEQUENCE=yes&I_THRESH=&LINE_LENGTH=60&MASK_CHAR=2&MASK_COLOR=1&NEW_VIEW=yes&NUM_OVERVIEW=100&PAGE=Proteins&QUERY_INDEX=0&QUERY_NUMBER=0&RESULTS_PAGE_TARGET=&RID=CDAED3D701R&SHOW_LINKOUT=yes&SHOW_OVERVIEW=yes&SORTED_DESCR_SEQS=ref\|WP_128886473.1\|,ref\|WP_156687440.1\|,ref\|WP_036470126.1\|,ref\|WP_065473763.1\|,ref\|WP_068157910.1\|,ref\|WP_085671006.1\|,ref\|WP_158017402.1\|,ref\|WP_069419255.1\|,ref\|WP_066939980.1\|,ref\|WP_211695689.1\|,ref\|WP_071023730.1\|,ref\|WP_067385316.1\|,ref\|WP_191498542.1\|,ref\|WP_083074451.1\|,ref\|WP_036402388.1\|,ref\|WP_180914149.1\|,ref\|WP_085263439.1\|,ref\|WP_144955623.1\|,ref\|WP_068117837.1\|,ref\|WP_085076954.1\|,ref\|WP_134429179.1\|,ref\|WP_120793264.1\|,ref\|WP_221367621.1\|,ref\|WP_062538994.1\|,gb\|OBK77433.1\|,gb\|KUI02473.1\|,ref\|WP_116540331.1\|,ref\|WP_119607974.1\|,ref\|WP_011779057.1\|,ref\|WP_099038968.1\|,gb\|MCB1287946.1\|,ref\|WP_066980105.1\|,ref\|WP_043410932.1\|,ref\|WP_083121049.1\|,ref\|WP_085108950.1\|,ref\|WP_163749624.1\|,ref\|WP_219066595.1\|,ref\|WP_143820166.1\|,ref\|WP_067396639.1\|,ref\|WP_240262914.1\|,ref\|WP_211156151.1\|,ref\|WP_085302105.1\|,ref\|WP_067331443.1\|,ref\|WP_036391701.1\|,gb\|MBX7456299.1\|,ref\|WP_064884554.1\|,ref\|WP_209922861.1\|,ref\|WP_083735722.1\|,ref\|WP_144285893.1\|,ref\|WP_155915580.1\|,ref\|WP_079923628.1\|,ref\|WP_090355981.1\|,ref\|WP_136910491.1\|,ref\|WP_140691504.1\|,ref\|WP_135361585.1\|,ref\|WP_208963521.1\|,ref\|WP_163734451.1\|,emb\|CKV23880.1\|,ref\|WP_083046484.1\|,ref\|WP_085161654.1\|,ref\|WP_075512658.1\|,ref\|WP_072516333.1\|,gb\|MBW0019183.1\|,ref\|WP_113964043.1\|,ref\|WP_065142642.1\|,gb\|KAA8964603.1\|,ref\|WP_085200206.1\|,gb\|MBS1692777.1\|,ref\|WP_085236188.1\|,ref\|WP_178357361.1\|,ref\|WP_159230910.1\|,ref\|WP_163686705.1\|,gb\|MBV9720335.1\|,ref\|WP_083149223.1\|,ref\|WP_163759712.1\|,gb\|RAU95435.1\|,) | **Sequence** [**Ident**](https://blast.ncbi.nlm.nih.gov/Blast.cgi?CMD=Get&ADV_VIEW=yes&ADV_VIEW=on&ALIGNMENTS=100&ALIGNMENT_VIEW=Pairwise&CDD_RID=CDAEDX7U01R&CDD_SEARCH_STATE=0&CONFIG_DESCR=ClustMemNbr,ClustComn,Ds,Sc,Ms,Ts,Cov,Eval,Idnt,AccLen,Acc&DATABASE_SORT=0&DESCRIPTIONS=100&DYNAMIC_FORMAT=on&FIRST_QUERY_NUM=0&FORMAT_NUM_ORG=1&FORMAT_OBJECT=Alignment&FORMAT_PAGE_TARGET=&FORMAT_TYPE=HTML&GET_SEQUENCE=yes&I_THRESH=&LINE_LENGTH=60&MASK_CHAR=2&MASK_COLOR=1&NEW_VIEW=yes&NUM_OVERVIEW=100&PAGE=Proteins&QUERY_INDEX=0&QUERY_NUMBER=0&RESULTS_PAGE_TARGET=&RID=CDAED3D701R&SHOW_LINKOUT=yes&SHOW_OVERVIEW=yes&SORTED_DESCR_SEQS=ref\|WP_128886473.1\|,ref\|WP_156687440.1\|,ref\|WP_036470126.1\|,ref\|WP_065473763.1\|,ref\|WP_068157910.1\|,ref\|WP_085671006.1\|,ref\|WP_158017402.1\|,ref\|WP_069419255.1\|,ref\|WP_066939980.1\|,ref\|WP_211695689.1\|,ref\|WP_071023730.1\|,ref\|WP_067385316.1\|,ref\|WP_191498542.1\|,ref\|WP_083074451.1\|,ref\|WP_036402388.1\|,ref\|WP_180914149.1\|,ref\|WP_085263439.1\|,ref\|WP_144955623.1\|,ref\|WP_068117837.1\|,ref\|WP_085076954.1\|,ref\|WP_134429179.1\|,ref\|WP_120793264.1\|,ref\|WP_221367621.1\|,ref\|WP_062538994.1\|,gb\|OBK77433.1\|,gb\|KUI02473.1\|,ref\|WP_116540331.1\|,ref\|WP_119607974.1\|,ref\|WP_011779057.1\|,ref\|WP_099038968.1\|,gb\|MCB1287946.1\|,ref\|WP_066980105.1\|,ref\|WP_043410932.1\|,ref\|WP_083121049.1\|,ref\|WP_085108950.1\|,ref\|WP_163749624.1\|,ref\|WP_219066595.1\|,ref\|WP_143820166.1\|,ref\|WP_067396639.1\|,ref\|WP_240262914.1\|,ref\|WP_211156151.1\|,ref\|WP_085302105.1\|,ref\|WP_067331443.1\|,ref\|WP_036391701.1\|,gb\|MBX7456299.1\|,ref\|WP_064884554.1\|,ref\|WP_209922861.1\|,ref\|WP_083735722.1\|,ref\|WP_144285893.1\|,ref\|WP_155915580.1\|,ref\|WP_079923628.1\|,ref\|WP_090355981.1\|,ref\|WP_136910491.1\|,ref\|WP_140691504.1\|,ref\|WP_135361585.1\|,ref\|WP_208963521.1\|,ref\|WP_163734451.1\|,emb\|CKV23880.1\|,ref\|WP_083046484.1\|,ref\|WP_085161654.1\|,ref\|WP_075512658.1\|,ref\|WP_072516333.1\|,gb\|MBW0019183.1\|,ref\|WP_113964043.1\|,ref\|WP_065142642.1\|,gb\|KAA8964603.1\|,ref\|WP_085200206.1\|,gb\|MBS1692777.1\|,ref\|WP_085236188.1\|,ref\|WP_178357361.1\|,ref\|WP_159230910.1\|,ref\|WP_163686705.1\|,gb\|MBV9720335.1\|,ref\|WP_083149223.1\|,ref\|WP_163759712.1\|,gb\|RAU95435.1\|,)**ity (%)** | **N. Residues** |
| --- | --- | --- | --- | --- |
| [WP_128886473.1](https://www.ncbi.nlm.nih.gov/protein/WP_128886473.1?report=genbank&log$=prottop&blast_rank=1&RID=CDAED3D701R) | [Mycobacterium tuberculosis](https://blast.ncbi.nlm.nih.gov/Blast.cgi#alnHdr_WP_128886473) | 97% | 99.84% | 647 |
| WP_036470126.1 | [Mycobacterium triplex](https://blast.ncbi.nlm.nih.gov/Blast.cgi#alnHdr_WP_036470126) | 97% | 86.08% | 641 |
| [WP_065473763.1](https://www.ncbi.nlm.nih.gov/protein/WP_065473763.1?report=genbank&log$=prottop&blast_rank=4&RID=CDAED3D701R) | [Mycobacterium malmoense](https://blast.ncbi.nlm.nih.gov/Blast.cgi#alnHdr_WP_065473763) | 97% | 86.30% | 644 |
| [WP_068157910.1](https://www.ncbi.nlm.nih.gov/protein/WP_068157910.1?report=genbank&log$=prottop&blast_rank=5&RID=CDAED3D701R) | [Mycobacterium kubicae](https://blast.ncbi.nlm.nih.gov/Blast.cgi#alnHdr_WP_068157910) | 97% | 85.60% | 641 |
| [WP_085671006.1](https://www.ncbi.nlm.nih.gov/protein/WP_085671006.1?report=genbank&log$=prottop&blast_rank=6&RID=CDAED3D701R) | [Mycobacterium szulgai](https://blast.ncbi.nlm.nih.gov/Blast.cgi#alnHdr_WP_085671006) | 97% | 85.94% | 646 |
| [WP_158017402.1](https://www.ncbi.nlm.nih.gov/protein/WP_158017402.1?report=genbank&log$=prottop&blast_rank=7&RID=CDAED3D701R) | [Mycobacterium basiliense](https://blast.ncbi.nlm.nih.gov/Blast.cgi#alnHdr_WP_158017402) | 97% | 87.26% | 655 |
| [WP_069419255.1](https://www.ncbi.nlm.nih.gov/protein/WP_069419255.1?report=genbank&log$=prottop&blast_rank=8&RID=CDAED3D701R) | [Mycobacterium intermedium](https://blast.ncbi.nlm.nih.gov/Blast.cgi#alnHdr_WP_069419255) | 97% | 87.18% | 645 |
| [WP_211695689.1](https://www.ncbi.nlm.nih.gov/protein/WP_211695689.1?report=genbank&log$=prottop&blast_rank=10&RID=CDAED3D701R) | [Mycobacterium spongiae](https://blast.ncbi.nlm.nih.gov/Blast.cgi#alnHdr_WP_211695689) | 97% | 84.91% | 647 |
| [WP_071023730.1](https://www.ncbi.nlm.nih.gov/protein/WP_071023730.1?report=genbank&log$=prottop&blast_rank=11&RID=CDAED3D701R) | [Mycobacterium talmoniae](https://blast.ncbi.nlm.nih.gov/Blast.cgi#alnHdr_WP_071023730) | 97% | 85.02% | 643 |
| [WP_191498542.1](https://www.ncbi.nlm.nih.gov/protein/WP_191498542.1?report=genbank&log$=prottop&blast_rank=13&RID=CDAED3D701R) | [Mycobacterium simulans](https://blast.ncbi.nlm.nih.gov/Blast.cgi#alnHdr_WP_191498542) | 97% | 87.11% | 647 |
| [WP_085263439.1](https://www.ncbi.nlm.nih.gov/protein/WP_085263439.1?report=genbank&log$=prottop&blast_rank=17&RID=CDAED3D701R) | [Mycolicibacter longobardus](https://blast.ncbi.nlm.nih.gov/Blast.cgi#alnHdr_WP_085263439) | 97% | 83.60% | 643 |
| [WP_144955623.1](https://www.ncbi.nlm.nih.gov/protein/WP_144955623.1?report=genbank&log$=prottop&blast_rank=18&RID=CDAED3D701R) | [Mycobacterium helveticum](https://blast.ncbi.nlm.nih.gov/Blast.cgi#alnHdr_WP_144955623) | 97% | 83.10% | 650 |
| [WP_085076954.1](https://www.ncbi.nlm.nih.gov/protein/WP_085076954.1?report=genbank&log$=prottop&blast_rank=20&RID=CDAED3D701R) | [Mycobacterium palustre](https://blast.ncbi.nlm.nih.gov/Blast.cgi#alnHdr_WP_085076954) | 97% | 84.02% | 642 |
| [WP_134429179.1](https://www.ncbi.nlm.nih.gov/protein/WP_134429179.1?report=genbank&log$=prottop&blast_rank=21&RID=CDAED3D701R) | [Mycobacterium ulcerans](https://blast.ncbi.nlm.nih.gov/Blast.cgi#alnHdr_WP_134429179) | 97% | 83.89% | 644 |
| [WP_062538994.1](https://www.ncbi.nlm.nih.gov/protein/WP_062538994.1?report=genbank&log$=prottop&blast_rank=24&RID=CDAED3D701R) | [Mycobacterium celatum](https://blast.ncbi.nlm.nih.gov/Blast.cgi#alnHdr_WP_062538994) | 97% | 82.75% | 642 |
| [WP_116540331.1](https://www.ncbi.nlm.nih.gov/protein/WP_116540331.1?report=genbank&log$=prottop&blast_rank=27&RID=CDAED3D701R) | [Mycobacterium uberis](https://blast.ncbi.nlm.nih.gov/Blast.cgi#alnHdr_WP_116540331) | 97% | 81.59% | 656 |
| [**WP_119607974.1**](https://www.ncbi.nlm.nih.gov/protein/WP_119607974.1?report=genbank&log$=prottop&blast_rank=28&RID=CDAED3D701R) | [**Mycobacterium leprae**](https://blast.ncbi.nlm.nih.gov/Blast.cgi#alnHdr_WP_119607974) | **97%** | **80.97%** | **656** |
| [WP_099038968.1](https://www.ncbi.nlm.nih.gov/protein/WP_099038968.1?report=genbank&log$=prottop&blast_rank=30&RID=CDAED3D701R) | [Mycobacterium neglectum](https://blast.ncbi.nlm.nih.gov/Blast.cgi#alnHdr_WP_099038968) | 97% | 81.42% | 645 |
| [WP_043410932.1](https://www.ncbi.nlm.nih.gov/protein/WP_043410932.1?report=genbank&log$=prottop&blast_rank=33&RID=CDAED3D701R) | [Mycobacterium rufum](https://blast.ncbi.nlm.nih.gov/Blast.cgi#alnHdr_WP_043410932) | 97% | 79.97% | 642 |
| [WP_083121049.1](https://www.ncbi.nlm.nih.gov/protein/WP_083121049.1?report=genbank&log$=prottop&blast_rank=34&RID=CDAED3D701R) | [Mycolicibacterium rhodesiae](https://blast.ncbi.nlm.nih.gov/Blast.cgi#alnHdr_WP_083121049) | 97% | 80.70% | 641 |
| [WP_085108950.1](https://www.ncbi.nlm.nih.gov/protein/WP_085108950.1?report=genbank&log$=prottop&blast_rank=35&RID=CDAED3D701R) | [Mycolicibacillus trivialis](https://blast.ncbi.nlm.nih.gov/Blast.cgi#alnHdr_WP_085108950) | 97% | 80.38% | 646 |
| [WP_163749624.1](https://www.ncbi.nlm.nih.gov/protein/WP_163749624.1?report=genbank&log$=prottop&blast_rank=36&RID=CDAED3D701R) | [Mycolicibacterium helvum](https://blast.ncbi.nlm.nih.gov/Blast.cgi#alnHdr_WP_163749624) | 97% | 79.43% | 646 |
| [WP_240262914.1](https://www.ncbi.nlm.nih.gov/protein/WP_240262914.1?report=genbank&log$=prottop&blast_rank=40&RID=CDAED3D701R) | [Mycobacterium paraterrae](https://blast.ncbi.nlm.nih.gov/Blast.cgi#alnHdr_WP_240262914) | 97% | 76.81% | 640 |
| [WP_211156151.1](https://www.ncbi.nlm.nih.gov/protein/WP_211156151.1?report=genbank&log$=prottop&blast_rank=41&RID=CDAED3D701R) | [Mycolicibacterium septicum](https://blast.ncbi.nlm.nih.gov/Blast.cgi#alnHdr_WP_211156151) | 97% | 77.19% | 651 |
| [WP_085302105.1](https://www.ncbi.nlm.nih.gov/protein/WP_085302105.1?report=genbank&log$=prottop&blast_rank=42&RID=CDAED3D701R) | [Mycobacterium koreense](https://blast.ncbi.nlm.nih.gov/Blast.cgi#alnHdr_WP_085302105) | 97% | 77.71% | 646 |
| [MBX7456299.1](https://www.ncbi.nlm.nih.gov/protein/MBX7456299.1?report=genbank&log$=prottop&blast_rank=45&RID=CDAED3D701R) | [Mycolicibacterium sp. 3033](https://blast.ncbi.nlm.nih.gov/Blast.cgi#alnHdr_MBX7456299) | 97% | 75.82% | 646 |
| [WP_209922861.1](https://www.ncbi.nlm.nih.gov/protein/WP_209922861.1?report=genbank&log$=prottop&blast_rank=47&RID=CDAED3D701R) | [Mycolicibacterium lutetiense](https://blast.ncbi.nlm.nih.gov/Blast.cgi#alnHdr_WP_209922861) | 97% | 77.46% | 650 |
| [WP_140691504.1](https://www.ncbi.nlm.nih.gov/protein/WP_140691504.1?report=genbank&log$=prottop&blast_rank=54&RID=CDAED3D701R) | [Mycobacterium hodleri](https://blast.ncbi.nlm.nih.gov/Blast.cgi#alnHdr_WP_140691504) | 97% | 75.08% | 648 |
| [WP_085161654.1](https://www.ncbi.nlm.nih.gov/protein/WP_085161654.1?report=genbank&log$=prottop&blast_rank=60&RID=CDAED3D701R) | [Mycobacterium lacus](https://blast.ncbi.nlm.nih.gov/Blast.cgi#alnHdr_WP_085161654) | 97% | 86.57% | 644 |
| [WP_075512658.1](https://www.ncbi.nlm.nih.gov/protein/WP_075512658.1?report=genbank&log$=prottop&blast_rank=61&RID=CDAED3D701R) | [Mycobacterium ostraviense](https://blast.ncbi.nlm.nih.gov/Blast.cgi#alnHdr_WP_075512658) | 97% | 88.21% | 647 |
| [WP_113964043.1](https://www.ncbi.nlm.nih.gov/protein/WP_113964043.1?report=genbank&log$=prottop&blast_rank=64&RID=CDAED3D701R) | [Mycobacterium shimoidei](https://blast.ncbi.nlm.nih.gov/Blast.cgi#alnHdr_WP_113964043) | 97% | 83.54% | 640 |
| [WP_065142642.1](https://www.ncbi.nlm.nih.gov/protein/WP_065142642.1?report=genbank&log$=prottop&blast_rank=65&RID=CDAED3D701R) | [Mycobacterium asiaticum](https://blast.ncbi.nlm.nih.gov/Blast.cgi#alnHdr_WP_065142642) | 97% | 83.96% | 649 |
| [WP_085200206.1](https://www.ncbi.nlm.nih.gov/protein/WP_085200206.1?report=genbank&log$=prottop&blast_rank=67&RID=CDAED3D701R) | [Mycobacterium fragae](https://blast.ncbi.nlm.nih.gov/Blast.cgi#alnHdr_WP_085200206) | 97% | 83.57% | 644 |
| [WP_085236188.1](https://www.ncbi.nlm.nih.gov/protein/WP_085236188.1?report=genbank&log$=prottop&blast_rank=69&RID=CDAED3D701R) | [Mycobacterium conspicuum](https://blast.ncbi.nlm.nih.gov/Blast.cgi#alnHdr_WP_085236188) | 97% | 82.28% | 641 |
| [WP_163686705.1](https://www.ncbi.nlm.nih.gov/protein/WP_163686705.1?report=genbank&log$=prottop&blast_rank=72&RID=CDAED3D701R) | [Mycolicibacterium gadium](https://blast.ncbi.nlm.nih.gov/Blast.cgi#alnHdr_WP_163686705) | 97% | 81.89% | 646 |
| [WP_163759712.1](https://www.ncbi.nlm.nih.gov/protein/WP_163759712.1?report=genbank&log$=prottop&blast_rank=75&RID=CDAED3D701R) | [Mycobacterium botniense](https://blast.ncbi.nlm.nih.gov/Blast.cgi#alnHdr_WP_163759712) | 97% | 81.55% | 647 |
| [RAU95435.1](https://www.ncbi.nlm.nih.gov/protein/RAU95435.1?report=genbank&log$=prottop&blast_rank=76&RID=CDAED3D701R) | [Mycolicibacter senuensis](https://blast.ncbi.nlm.nih.gov/Blast.cgi#alnHdr_RAU95435) | 97% | 80.47% | 649 |
| [WP_014208752.1](https://www.ncbi.nlm.nih.gov/protein/WP_014208752.1?report=genbank&log$=prottop&blast_rank=77&RID=CDAED3D701R) | [Mycolicibacterium rhodesiae](https://blast.ncbi.nlm.nih.gov/Blast.cgi#alnHdr_WP_014208752) | 97% | 80.44% | 643 |
| [WP_097942061.1](https://www.ncbi.nlm.nih.gov/protein/WP_097942061.1?report=genbank&log$=prottop&blast_rank=81&RID=CDAED3D701R) | [Mycolicibacterium agri](https://blast.ncbi.nlm.nih.gov/Blast.cgi#alnHdr_WP_097942061) | 97% | 80.28% | 644 |
| [WP_085195702.1](https://www.ncbi.nlm.nih.gov/protein/WP_085195702.1?report=genbank&log$=prottop&blast_rank=84&RID=CDAED3D701R) | [Mycobacterium xenopi](https://blast.ncbi.nlm.nih.gov/Blast.cgi#alnHdr_WP_085195702) | 97% | 80.06% | 641 |
| [OLO99293.1](https://www.ncbi.nlm.nih.gov/protein/OLO99293.1?report=genbank&log$=prottop&blast_rank=94&RID=CDAED3D701R) | [Mycobacterium porcinum](https://blast.ncbi.nlm.nih.gov/Blast.cgi#alnHdr_OLO99293) | 97% | 78.16% | 635 |

**BlastP was run against mycobacteria (taxid 85007)*

**Table S2**. List of primers used in this study.

| **Primer name** | **Primer sequence** |
| --- | --- |
| *HtpG*_Mtb_ *fw* | 5’-CATATGAACGCCCATGTCGAGCAGTTG-3 |
| *HtpG*_Mtb_ *rv* | 5’-GAATTCGGCAAGGTACGCGCGAGACGTTC-3’ |
| *HtpG*_Mtb_*_N fw* | 5′-CATATGAACGCCCATGTCGAGCAGTTG-3′ |
| *HtpG*_Mtb_*_N rv* | 5′-AAGCTTCTTCATCGAGTTGAGGGT-3′ |
| *HtpG*_Mtb_*_M fw* | 5′-CATATGAACCTGGTCAAGAAATAC-3′ |
| *HtpG*_Mtb_*_M rv* | 5′-AAGCTTGGCGTAGAAGATCTGTTG-3′ |
| *HtpG*_Mtb_*_C fw* | 5’-CATATGGAGGGACTGCTGTCAGAC-3’ |
| *HtpG*_Mtb_*_C rv* | 5’-AAGCTTCAAGGTACGCGCGAGACGTTC-3’ |
| *HtpG*_Mtb_*_MC fw* | 5′-GAAGGAGATATACATATGAACCTGGTCAAGAAATACTCC-3′ |
| *HtpG*_Mtb_*_MC rv* | 5′-GAATTCGGCAAGGTACGCGCGAGACGTTC-3′ |
| *HtpG*_Mtb_*_C^F635E^ fw* | 5’-cgaggatccggcgaggGAAgccgagctgctggccg-3’ |
| *HtpG*_Mtb_*_C^F635E^ rv* | 5’-cggccagcagctcggcTTCcctcgccggatcctcg-3’ |
| *HtpG*_Mtb_*_C^Triple mutants^ rv1* | 5’-ttcggcCGCCGCctcggcCGCcctcgccggatcctcgag-3’ |
| *HtpG*_Mtb_*_C^Triple mutants^ rv2* | 5’-CAAGGTACGCGCGAGACGttcggcCGCCGCctcgg-3’ |
| *ESAT-6 fw* | 5′-AAGCTTATGACAGAGCAGCAGTGGAAT-3′ |
| *ESAT-6 rv* | 5′-CTCGAGTGCGAACATCCCAGTGACGTT-3′ |

**Table S3.** Prediction of MHCII binding epitopes, computed using NetMHCII4.1 [1]. Only epitopes with IC50 < 50 nM are reported. N, M and C domains are coloured white, grey and orange, respectively.

| **Residue MHC Peptide Score_EL Rank_EL Score_BA Affinity(nM) %Rank** |
| --- |
| **HLA-DR**  632 HLA-DRB1_0101 PARFAELLAERLART 0.980246 0.04 0.850571 5.04 0.11  277 HLA-DRB1_0101 PLEIIAMKAEGTFEY 0.927697 0.29 0.825021 6.64 0.27  88 HLA-DRB1_0101 REEVVDLIGTLAKSG 0.880262 0.50 0.705664 24.16 2.71  241 HLA-DRB1_0101 TETLNSMKALWARPK 0.854988 0.62 0.814585 7.43 0.36  565 HLA-DRB1_0101 LARIYRASGQEVPVG 0.779872 0.97 0.739239 16.80 1.66  277 HLA-DRB1_0102 PLEIIAMKAEGTFEY 0.976929 0.04 0.735766 17.44 0.05  88 HLA-DRB1_0102 REEVVDLIGTLAKSG 0.961380 0.12 0.646559 45.79 0.72  241 HLA-DRB1_0102 TETLNSMKALWARPK 0.938321 0.24 0.745781 15.65 0.04  565 HLA-DRB1_0102 LARIYRASGQEVPVG 0.932525 0.27 0.655342 41.64 0.60  632 HLA-DRB1_0102 PARFAELLAERLART 0.807219 0.95 0.651964 43.19 0.64  132 HLA-DRB1_0301 SSFMVADKVQLLTRK 0.802446 0.58 0.720921 20.48 0.03  353 HLA-DRB1_0301 REILQQDRQIKAIRR 0.795016 0.61 0.659369 39.87 0.19  132 HLA-DRB1_0305 SSFMVADKVQLLTRK 0.628211 0.77 0.655874 41.40 0.01  29 HLA-DRB1_0404 KDAFLRELISNASDA 0.821082 0.62 0.683509 30.70 0.21  203 HLA-DRB1_0701 VKKYSDFIAWPIRMD 0.758551 0.62 0.678476 32.42 0.92  565 HLA-DRB1_0901 LARIYRASGQEVPVG 0.865542 0.12 0.660605 39.34 0.56  564 HLA-DRB1_0901 ALARIYRASGQEVPV 0.786760 0.34 0.674613 33.81 0.40  632 HLA-DRB1_1001 PARFAELLAERLART 0.827655 0.41 0.775514 11.35 0.25  277 HLA-DRB1_1001 PLEIIAMKAEGTFEY 0.782705 0.61 0.715714 21.67 1.21  241 HLA-DRB1_1001 TETLNSMKALWARPK 0.721137 0.96 0.726246 19.34 0.94  137 HLA-DRB1_1101 ADKVQLLTRKAGESA 0.962217 0.11 0.642712 47.74 0.99  562 HLA-DRB1_1101 TPALARIYRASGQEV 0.931424 0.26 0.673839 34.09 0.56  358 HLA-DRB1_1101 QDRQIKAIRRRLTKK 0.915479 0.33 0.812548 7.60 0.00  195 HLA-DRB1_1101 SEWKIRNLVKKYSDF 0.828901 0.72 0.713986 22.08 0.21  137 HLA-DRB1_1104 ADKVQLLTRKAGESA 0.986068 0.00 0.728214 18.93 0.33  562 HLA-DRB1_1104 TPALARIYRASGQEV 0.969054 0.07 0.736586 17.29 0.26  359 HLA-DRB1_1104 DRQIKAIRRRLTKKV 0.960354 0.11 0.855158 4.79 0.00  195 HLA-DRB1_1104 SEWKIRNLVKKYSDF 0.925708 0.25 0.791065 9.59 0.04  538 HLA-DRB1_1104 VKEVRLSTRLTESPA 0.767768 0.99 0.658117 40.41 1.45  359 HLA-DRB1_1301 DRQIKAIRRRLTKKV 0.898244 0.12 0.793979 9.29 0.00  196 HLA-DRB1_1301 EWKIRNLVKKYSDFI 0.717123 0.97 0.669722 35.64 0.72  580 HLA-DRB1_1302 KRILELNPSHPLVTG 0.695800 0.27 0.782141 10.56 0.06  556 HLA-DRB5_0101 TDAFGMTPALARIYR 0.821184 0.11 0.780394 10.76 0.10  580 HLA-DRB3_0202 KRILELNPSHPLVTG 0.844437 0.13 0.749393 15.05 0.13  132 HLA-DRB3_0101 SSFMVADKVQLLTRK 0.677992 0.40 0.731786 18.21 0.12  632 HLA-DRB1_1601 PARFAELLAERLART 0.817809 0.33 0.628373 45.75 0.57  125 HLA-DRB1_1501 QFGIGFYSSFMVADK 0.926789 0.12 0.784496 10.30 0.02  199 HLA-DRB1_1501 IRNLVKKYSDFIAWP 0.921646 0.13 0.688182 29.19 0.36  413 HLA-DRB1_1501 LLGISSFVSTYSEEE 0.671316 0.94 0.674859 33.72 0.48  580 HLA-DRB1_1402 KRILELNPSHPLVTG 0.609383 0.57 0.623214 58.95 0.09 |
| **HLA-DP**  487 HLA-DPA10103-DPB10201 VPEFDGKPLQSVAKG 0.932578 0.04 0.531381 49.23 3.92  462 HLA-DPA10103-DPB10201 LEAFKAKGYEVLLLT 0.895231 0.09 0.772524 11.72 0.06  288 HLA-DPA10103-DPB10201 TFEYQALLFIPSHAP 0.682907 0.47 0.741204 16.45 0.13  288 HLA-DPA10103-DPB10401 TFEYQALLFIPSHAP 0.427780 0.38 0.689611 28.74 0.30  462 HLA-DPA10103-DPB10401 LEAFKAKGYEVLLLT 0.277490 0.82 0.654762 41.90 0.52  288 HLA-DPA10103-DPB12301 TFEYQALLFIPSHAP 0.427780 0.38 0.689611 28.74 0.30 A-DPA10103-DPB12301 LEAFKAKGYEVLLLT 0.277490 0.82 0.654762 41.90 0.52 |

## 2 Supplementary Figures

**Supplementary Figure S1.** Prediction of B cell epitopes, according to Discotope [2].


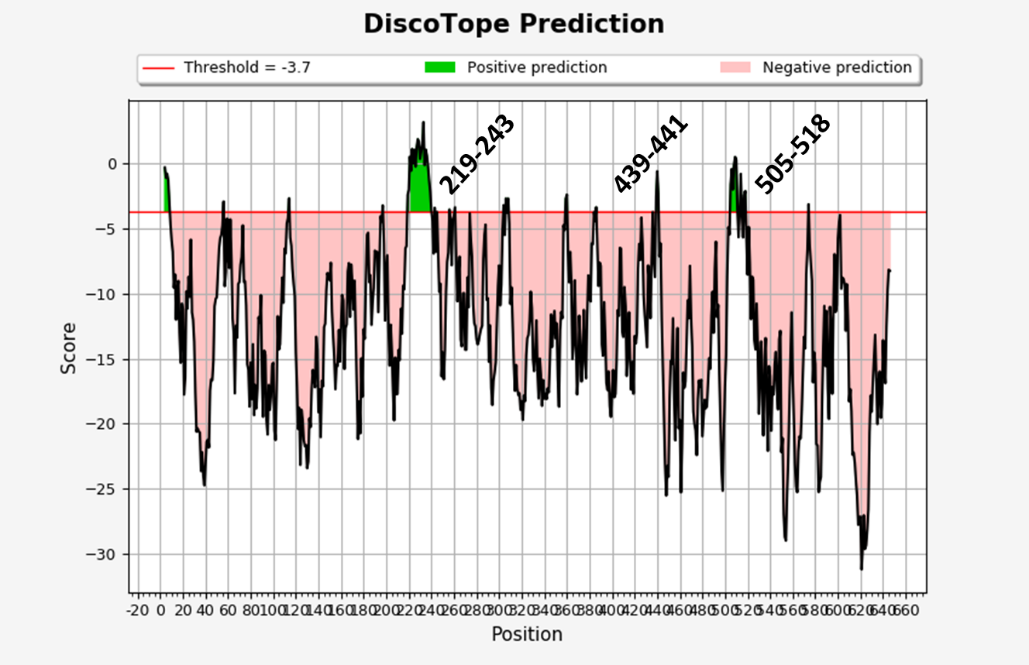


**Supplementary references**

1. Reynisson, B.; Alvarez, B.; Paul, S.; Peters, B.; Nielsen, M. NetMHCpan-4.1 and NetMHCIIpan-4.0: Improved Predictions of MHC Antigen Presentation by Concurrent Motif Deconvolution and Integration of MS MHC Eluted Ligand Data. *Nucleic Acids Res* **2020**, *48*, W449–W454, doi:10.1093/nar/gkaa379.

2. Kringelum, J.V.; Lundegaard, C.; Lund, O.; Nielsen, M. Reliable B Cell Epitope Predictions: Impacts of Method Development and Improved Benchmarking. *PLoS Comput Biol* **2012**, *8*, e1002829, doi:10.1371/journal.pcbi.1002829.
